# Global, Regional, and National Burden of Male Breast Cancer, 1990–2021: A Systematic Analysis for the Global Burden of Disease Study 2021

**DOI:** 10.1002/cam4.70632

**Published:** 2025-01-29

**Authors:** Long Wang, Ping Wen, Qing Shao, Dongping Jiang, Yulan Zhao, Xiaohua Zeng

**Affiliations:** ^1^ Department of Breast Cancer Center Chongqing University Cancer Hospital Chongqing China; ^2^ Department of Breast Cancer Center Chongqing University Cancer Hospital, School of Medicine, Chongqing University Chongqing China; ^3^ Department of Medical Insurance Chongqing University Cancer Hospital Chongqing China

**Keywords:** epidemiology, global burden of disease study 2021, male breast cancer, risk factor

## Abstract

**Background:**

Male breast cancer (MBC) is rare and often treated using evidence from female breast cancer (BC) trials due to limited male participation. Previous estimates lacked global coverage and completeness. We aimed to quantify the global MBC burden from 1990 to 2021 and evaluate its current status and trends.

**Methods:**

Based on the global burden of disease (GBD) database, we gathered and analyzed data on the incidence, death, and disability‐adjusted life years (DALYs) of MBC while utilizing age‐standardized rates (ASRs) as indicators for these measurements. Our study calculated the estimated annual percentage change (EAPC), aiming at measuring the average change in ASRs. Additionally, we evaluated the attributable risk factors (RFs) and trends of MBC across different regions and age groups worldwide.

**Results:**

In 2021, the global MBC age‐standardized incidence rates (ASIR), age‐standardized death rates (ASDR), and age‐standardized DALY rates (ASDALY) per 100,000 persons were 0.941 (95% UI, 0.605–1.155), 0.335 (95% UI, 0.232–0.409), and 9.157 (95% UI, 6.116–11.423), respectively. In comparison to 1990, these rates have increased by 2.212 (95% UI, 2.047–2.378), 0.664 (95% UI, 0.562–0.767), and 0.853 (95% UI, 0.750–0.956) respectively. In Uganda 2021, the ASIR and ASDR of MBC were the highest at 4.541 (95% UI, 3.028–6.808) and 3.510 (95% UI, 2.301–5.195) per 100,000 persons, respectively. Moreover, the burden of MBC exhibited an increase with age. Globally, dietary risk was the most important attributable RF for MBC deaths, with a death percentage of 11.690% (95% UI, −0.003%–24.838%), followed by alcohol use and tobacco.

**Conclusion:**

From 1990 to 2021, the ASIR, ASDR, and ASDALY of MBC have shown significant disparities and an increasing trend. Committing to healthy lifestyle choices, such as decreasing tobacco and alcohol consumption and making positive changes to dietary habits, can assist in reducing MBC risk. The development and execution of robust and effective public health policies are crucial for alleviating the global disease burden.

**Abbreviations**
ASDALYR: age‐standardized disability‐adjusted life year rate
ASDR: age‐standardized death rate
ASIR: age‐standardized incidence rate
ASRs: age‐standardized rates
BC: breast cancer
CI: confidence interval
DALYs: disability‐adjusted life years
EAPC: estimated annual percentage change
GBD: global burden of disease
ICD: International Classification of Diseases
IHME: Institute for Health Metrics and Evaluation
MBC: male breast cancer
RFs: risk factors
ST‐GPR: spatiotemporal gaussian process regression
UI: uncertainty interval

## 1 Introduction

Breast cancer (BC) has emerged as the most prominent neoplasm worldwide by being the most threatening tumor that poses a significant risk to the well‐being of females and has consequently garnered much attention [1]. Nevertheless, male breast cancer (MBC) has received less focus. Unlike female BC, MBC is an exceptionally uncommon cancer, representing below 1% of all BCs and 0.1% of all malignancies in males. Consequently, only a small number of males actively take the initiative in BC screening [2]. Breast screening surveys in normal medical visits are often limited to females in many countries, excluding men. This results in the detection of MBC at advanced stages, leading to a more unfavorable prognosis. The unique challenges faced by males with BC, including psychological impacts and social stigma, necessitate tailored support and awareness programs [3, 4]. Historically, BC has been predominantly viewed as a “women's disease,” which has contributed to a lack of awareness and research emphasis on MBC [5, 6]. The rarity of MBC, accounting for less than 1% of all BCs, has further led to its underrepresentation in research [7]. Sociocultural perceptions and stigma also play a significant role; men diagnosed with BC may face challenges to their masculinity and are often less likely to seek screening or medical advice, further exacerbating late diagnosis and poorer outcomes [8]. Addressing these issues is essential to improving early detection rates and treatment outcomes, ultimately reducing the global burden of MBC.

Recent studies have highlighted a globally elevating trend in MBC incidence and burden. This rising concern necessitates a comprehensive understanding of its epidemiology and associated risk factors (RFs). An 11‐year retrospective multicentric study conducted in the Czech Republic revealed that nearly 580 MBC cases were identified, with an estimated 45–65 new cases being diagnosed yearly [9]. Previous studies have demonstrated that the incidence of MBC increased in nearly two‐thirds of countries between 1990 and 2017, with significant rises observed primarily in Asia and North Africa. In contrast, most countries in Europe and North America saw either a decline or stabilization in MBC incidence rates [10]. Moreover, numerous studies particularly exclude males diagnosed with BC. For instance, Duma et al. have examined 426 therapeutic BC trials conducted between January 2000 and April 2017. Their analysis has revealed that 65% (*n* = 277) of these trials had enrollment criteria that specifically excluded males. Additionally, due to the infrequency of MBC, its recommended treatment guidelines are often derived from clinical trial data that mostly includes female BC patients [11]. Consequently, there is limited information on the epidemiology, RFs, and treatment strategies unique to MBC. Future research should prioritize this rare but significant group to better understand and manage their specific needs and challenges.

This study, conducted as part of global burden of disease (GBD) 2021 [12], presented data on the global distribution and trends in MBC burden from 1990 to 2021. To our knowledge, few long‐term global trends in the epidemiology of MBC have been reported. Here, we utilized the GBD database to analyze MBC incidence, death, and DALYs, with corresponding ASRs, from 1990 to 2021. We hope that this interpretation of GBD 2021 estimates for healthcare professionals will facilitate the development of new prevention and treatment approaches that can mitigate the health risks of MBC.

## 2 Materials and Methods

### 2.1 Data Acquisition and Download

The 2021 GBD study offers an extensive assessment of the health impacts associated with 371 diseases, injuries, and impairments, as well as 88 RFs. Supported by over 11,500 contributors across 164 countries, this evaluation analyzes global health and disease burden through thorough data collection, review, and analysis. The data sources for the GBD 2021 include epidemiological surveys, hospital records, vital registration systems, disease monitoring systems, and a wide range of academic papers and policy reports (https://ghdx.healthdata.org/gbd‐2021/sources). These data are standardized using the International Classification of Diseases (ICD) codes to ensure accuracy and comparability. To estimate cancer burden, advanced modeling tools such as DisMod‐MR and spatiotemporal Gaussian process regression (ST‐GPR) are employed. The data processing also includes adjustments for heterogeneity and bias, with uncertainty analysis conducted via Monte Carlo simulations [12, 13].

For this study, global MBC data from 1990 to 2021 were retrieved using the GBD Results Tool, provided by IHME (https://vizhub.healthdata.org/gbd‐results/) [14]. The data used in this analysis specifically included only MBC cases, with female BC patients excluded from the dataset. Three key aspects of the MBC burden were analyzed: the number of incident cases, deaths, and DALYs, along with their corresponding crude rates and age‐standardized rates (ASRs). Additionally, 95% uncertainty intervals (UI) were calculated for MBC incidence, deaths, and DALYs. The data were stratified by geographic regions, age groups, and RFs to provide a comprehensive analysis of MBC trends and burden over time.

### 2.2 Risk Factors Analysis

The GBD study systematically assesses the impact of 88 RFs on health outcomes globally. These RFs are categorized into behavioral, metabolic, and environmental groups. For MBC, key RFs include tobacco use, alcohol consumption, and dietary risks, all of which have been shown to significantly contribute to the development of BC in males. In this study, we focused on these three primary RFs to assess their impact on MBC burden over time.

### 2.3 Statistical Analysis

The MBC burden was described using the following main indicators: numbers of incidence, deaths, DALYs, and their corresponding ASRs. Each rate is reported per 100,000 population, along with 95% UI according to the GBD algorithm. The dynamics of MBC were analyzed by calculating estimated annual percentage changes (EAPCs) to identify temporal trends in the disease burden; 95% CIs of EAPCs were determined by linear modeling. If the upper limit of both an EAPC and its 95% CI is negative, its corresponding rate shows a decreasing trend; conversely, if the lower limit of both an EAPC and its 95% CI is positive, its corresponding rate shows an increasing trend. All calculations were conducted through R Studio, version 4.3.1 (R Project for Statistical Computing). The *p* values were two‐sided, considering *p* < 0.05 statistically significant.

## 3 Results

### 3.1 Global Burden

The global MBC incident cases reached 38827.302 (95% UI, 24650.482–47845.887) in 2021, compared with 9776.514 (95% UI, 8474.102–11224.790) cases in 2019. Throughout 1990–2021, the global MBC incident cases elevated by 2.971 times (95% UI, 1.610–4.016; Table 1). The corresponding global ASIR increased accordingly from 0.525 (95% UI, 0.460–0.603) in 1990 to 0.941 (95% UI, 0.605–1.155) in 2021; the EAPC was 2.212 (95% CI, 2.047–2.378; Table 2).

**TABLE 1:** Incident, death, DALY cases during 1990–2021, and percentage change for MBC from 1990 to 2021 globally and regionally.

	Incident cases (95% UI)			Deaths (95% UI)			DALYs (95% UI)		
Location	1990	2021	Percentage change in cases, 1990–2021	1990	2021	Percentage change in cases, 1990–2021	1990	2021	Percentage change in cases, 1990–2021
Global	9776.514 (8474.102–11224.790)	38827.302 (24650.482–47845.887)	2.971 (1.610–4.016)	4899.024 (4142.232–5895.072)	13274.114 (9074.300–16240.064)	1.710 (0.869–2.296)	144956.307 (121255.738–172477.024)	380916.567 (252900.074–476416.749)	1.628 (0.777–2.238)
Andean Latin America	16.266 (11.544–23.104)	88.495 (55.047–120.969)	4.440 (2.144–7.455)	12.065 (8.665–17.409)	43.231 (28.340–58.032)	2.583 (1.112–4.363)	366.644 (260.314–527.690)	1249.481 (797.893–1701.261)	2.408 (0.981–4.145)
Australasia	82.763 (69.851–96.690)	218.623 (175.932–268.690)	1.642 (1.011–2.465)	23.212 (20.459–26.191)	48.303 (39.648–58.275)	1.081 (0.659–1.616)	600.102 (524.574–681.256)	1148.125 (942.715–1383.259)	0.913 (0.524–1.390)
Caribbean	41.024 (34.932–49.356)	178.287 (148.588–214.775)	3.346 (2.485–4.428)	28.064 (23.544–34.720)	93.502 (77.223–111.364)	2.332 (1.642–3.190)	743.547 (623.690–932.223)	2439.195 (1998.805–2963.354)	2.280 (1.570–3.171)
Central Asia	18.233 (14.609–22.206)	79.576 (67.476–93.252)	3.364 (2.372–4.642)	10.673 (8.404–13.044)	38.141 (32.836–44.177)	2.574 (1.779–3.600)	328.704 (266.485–398.868)	1135.034 (964.850–1330.053)	2.453 (1.651–3.413)
Central Europe	314.242 (279.352–349.125)	901.342 (790.473–1014.937)	1.868 (1.496–2.343)	162.350 (144.115–181.094)	346.565 (303.865–383.533)	1.135 (0.861–1.435)	4347.120 (3857.763–4838.151)	8289.170 (7288.039–9239.379)	0.907 (0.664–1.169)
Central Latin America	53.456 (50.224–56.241)	313.282 (274.130–358.904)	4.861 (4.043–5.819)	37.154 (34.818–39.138)	159.935 (139.819–181.306)	3.305 (2.739–3.925)	1110.500 (1040.668–1169.013)	4430.665 (3892.193–5069.906)	2.990 (2.455–3.568)
Central sub‐Saharan Africa	82.336 (53.960–132.907)	225.260 (144.656–364.945)	1.736 (0.918–3.043)	67.698 (44.344–109.172)	158.034 (101.123–260.232)	1.334 (0.647–2.462)	2095.436 (1386.513–3347.598)	5092.082 (3265.782–8367.092)	1.430 (0.671–2.576)
East Asia	1943.392 (1332.437–2657.376)	17122.003 (6888.342–24713.833)	7.810 (2.921–13.368)	876.110 (604.676–1191.997)	3439.080 (1368.006–4948.593)	2.925 (0.792–5.463)	29733.977 (19896.948–40540.980)	110295.773 (42489.925–159208.325)	2.709 (0.703–5.125)
Eastern Europe	876.182 (810.751–935.332)	865.788 (738.622–980.969)	−0.012 (−0.133–0.118)	405.935 (374.942–433.360)	295.962 (252.615–338.861)	−0.271 (−0.369 to −0.160)	12101.446 (11172.278–12915.760)	8372.692 (7145.452–9607.647)	−0.308 (−0.400 to −0.202)
Eastern sub‐Saharan Africa	983.680 (751.727–1532.046)	2476.279 (1598.388–4501.435)	1.517 (0.733–2.479)	822.066 (629.593–1286.111)	1748.811 (1142.629–3196.076)	1.127 (0.473–1.921)	24353.413 (18487.954–37985.442)	52924.102 (34090.290–97872.761)	1.173 (0.493–2.070)
High‐income Asia Pacific	157.138 (139.452–177.459)	461.877 (398.993–528.196)	1.939 (1.480–2.504)	65.337 (59.074–71.578)	138.689 (125.986–149.860)	1.123 (0.884–1.374)	1720.635 (1527.162–1896.975)	2922.981 (2621.906–3238.990)	0.699 (0.493–0.956)
High‐income North America	2132.618 (2048.107–2205.108)	3570.251 (3378.625–3733.135)	0.674 (0.595–0.753)	404.449 (387.472–417.298)	652.787 (611.904–685.767)	0.614 (0.547–0.677)	11721.756 (11120.954–12399.514)	17502.033 (16365.059–18784.703)	0.493 (0.434–0.551)
North Africa and Middle East	297.893 (216.402–412.305)	1371.104 (1008.434–1874.872)	3.603 (1.833–5.306)	205.055 (148.798–288.271)	613.644 (452.459–839.196)	1.993 (0.891–3.103)	6080.434 (4365.925–8461.262)	17668.136 (13079.763–24021.786)	1.906 (0.827–2.964)
Oceania	1.958 (1.128–3.211)	7.387 (3.549–12.522)	2.772 (1.508–4.246)	1.422 (0.824–2.312)	4.859 (2.352–8.454)	2.416 (1.265–3.792)	45.571 (25.484–74.723)	154.077 (73.038–266.404)	2.381 (1.239–3.680)
South Asia	824.647 (647.605–1123.471)	4067.409 (2449.812–5161.404)	3.932 (1.772–5.682)	661.102 (519.499–915.067)	2587.911 (1568.129–3266.468)	2.915 (1.214–4.278)	19223.589 (15206.266–26114.227)	70243.551 (42413.706–90053.447)	2.654 (1.065–3.943)
Southeast Asia	300.773 (193.705–368.311)	1524.395 (801.622–1914.423)	4.068 (2.437–5.498)	216.868 (139.083–267.563)	837.843 (439.890–1048.971)	2.863 (1.627–4.004)	6638.333 (4164.047–8207.905)	24624.975 (12684.014–31266.664)	2.710 (1.487–3.820)
Southern Latin America	116.690 (96.291–140.448)	273.474 (225.433–322.432)	1.344 (0.905–1.941)	75.633 (62.479–91.970)	128.443 (107.291–149.499)	0.698 (0.371–1.119)	1988.026 (1651.510–2366.664)	3150.931 (2631.623–3635.697)	0.585 (0.323–0.968)
Southern sub‐Saharan Africa	69.469 (52.024–92.022)	233.564 (164.683–305.665)	2.362 (1.558–3.192)	49.248 (36.971–66.763)	140.782 (100.496–180.866)	1.859 (1.202–2.546)	1565.219 (1170.034–2043.179)	4346.198 (3083.390–5693.596)	1.777 (1.133–2.427)
Tropical Latin America	123.496 (114.161–132.543)	829.770 (763.089–898.687)	5.719 (5.131–6.342)	84.893 (78.636–90.907)	428.060 (392.737–458.972)	4.042 (3.631–4.433)	2495.701 (2316.664–2680.878)	11983.779 (11034.519–12824.485)	3.802 (3.419–4.183)
Western Europe	1127.230 (1049.380–1201.583)	3558.755 (3115.022–3955.629)	2.157 (1.833–2.533)	515.696 (482.440–545.442)	1048.476 (926.926–1151.827)	1.033 (0.848–1.220)	12605.237 (11800.931–13406.758)	23410.801 (20864.754–25976.552)	0.857 (0.684–1.040)
Western sub‐Saharan Africa	213.027 (123.820–330.596)	460.382 (297.052–837.432)	1.161 (0.413–2.211)	173.993 (101.737–267.870)	321.055 (209.916–577.545)	0.845 (0.201–1.702)	5090.918 (2962.592–7930.866)	9532.786 (6181.103–17502.795)	0.873 (0.221–1.779)

**TABLE 2:** Age‐standardized incidence rate (ASIR), age‐standardized death rate (ASDR), age‐standardized disability‐adjusted life year (ASDALY) per 100,000 population in 1990–2021, and estimated annual percentage change (EAPC) for MBC from 1990 to 2021 globally and regionally.

Location	ASIR (95% UI)			ASDR (95% UI)			ASDALYR (95% UI)		
	1990	2021	EPAC, 1990–2021	1990	2021	EPAC, 1990–2021	1990	2021	EPAC, 1990–2021
Global	0.525 (0.460–0.603)	0.941 (0.605–1.155)	2.212 (2.047–2.378)	0.282 (0.241–0.338)	0.335 (0.232–0.409)	0.664 (0.562–0.767)	7.386 (6.230–8.827)	9.157 (6.116–11.423)	0.853 (0.750–0.956)
Andean Latin America	0.155 (0.111–0.219)	0.304 (0.190–0.413)	2.937 (2.413–3.463)	0.120 (0.087–0.173)	0.153 (0.101–0.203)	1.421 (0.885–1.960)	3.275 (2.323–4.724)	4.201 (2.694–5.700)	1.426 (0.874–1.981)
Australasia	0.782 (0.665–0.903)	0.920 (0.741–1.135)	0.760 (0.506–1.015)	0.237 (0.208–0.267)	0.192 (0.158–0.231)	−0.400 (−0.673 to −0.127)	5.696 (5.007–6.441)	4.867 (3.998–5.875)	−0.227 (−0.507–0.054)
Caribbean	0.330 (0.281–0.396)	0.706 (0.588–0.850)	2.257 (1.681–2.836)	0.235 (0.198–0.289)	0.373 (0.308–0.444)	1.257 (0.768–1.748)	5.807 (4.872–7.266)	9.585 (7.859–11.650)	1.378 (0.908–1.851)
Central Asia	0.091 (0.072–0.111)	0.218 (0.188–0.253)	4.444 (3.557–5.339)	0.059 (0.045–0.072)	0.117 (0.102–0.135)	3.792 (2.929–4.662)	1.547 (1.229–1.880)	2.937 (2.525–3.411)	3.489 (2.675–4.310)
Central Europe	0.491 (0.438–0.546)	0.972 (0.854–1.090)	2.182 (1.837–2.527)	0.273 (0.243–0.306)	0.376 (0.330–0.416)	1.027 (0.769–1.285)	6.659 (5.929–7.392)	9.000 (7.899–10.018)	0.922 (0.640–1.205)
Central Latin America	0.127 (0.120–0.134)	0.267 (0.234–0.305)	1.640 (0.837–2.450)	0.094 (0.088–0.099)	0.140 (0.123–0.159)	0.516 (−0.258–1.296)	2.474 (2.318–2.602)	3.695 (3.253–4.228)	0.523 (−0.243–1.294)
Central sub‐Saharan Africa	0.762 (0.494–1.207)	0.852 (0.551–1.385)	0.214 (−0.021–0.450)	0.673 (0.437–1.059)	0.666 (0.427–1.145)	−0.185 (−0.368 to −0.002)	17.560 (11.580–28.170)	16.952 (10.822–28.075)	−0.268 (−0.452 to −0.083)
East Asia	0.402 (0.284–0.552)	1.567 (0.637–2.245)	6.227 (5.457–7.003)	0.199 (0.139–0.269)	0.323 (0.134–0.463)	2.896 (2.314–3.482)	5.923 (4.090–8.012)	10.097 (3.931–14.488)	3.101 (2.493–3.713)
Eastern Europe	0.859 (0.795–0.919)	0.634 (0.542–0.716)	−1.253 (−1.551 to −0.954)	0.424 (0.392–0.455)	0.222 (0.190–0.253)	−2.460 (−2.806 to −2.112)	11.273 (10.401–12.058)	6.082 (5.214–6.940)	−2.384 (−2.753 to −2.013)
Eastern sub‐Saharan Africa	2.661 (2.030–4.148)	3.046 (2.009–5.461)	0.279 (0.224–0.333)	2.384 (1.827–3.738)	2.386 (1.601–4.286)	−0.154 (−0.216 to −0.092)	59.688 (45.592–94.105)	57.763 (37.899–105.577)	−0.282 (−0.345 to −0.220)
High‐income Asia Pacific	0.181 (0.162–0.205)	0.229 (0.199–0.261)	0.293 (−0.172–0.760)	0.082 (0.074–0.089)	0.063 (0.058–0.069)	−1.384 (−1.746 to −1.020)	1.931 (1.721–2.121)	1.493 (1.339–1.666)	−1.388 (−1.761 to −1.014)
High‐income North America	1.429 (1.372–1.477)	1.227 (1.162–1.283)	−0.967 (−1.298 to −0.635)	0.277 (0.264–0.286)	0.219 (0.205–0.230)	−1.197 (−1.469 to −0.924)	7.921 (7.515–8.383)	6.080 (5.684–6.526)	−1.245 (−1.536 to −0.953)
North Africa and Middle East	0.344 (0.253–0.477)	0.579 (0.423–0.794)	1.783 (1.717–1.850)	0.256 (0.186–0.358)	0.284 (0.210–0.389)	0.301 (0.247–0.356)	6.478 (4.697–9.042)	7.033 (5.208–9.553)	0.246 (0.190–0.302)
Oceania	0.123 (0.071–0.198)	0.180 (0.087–0.305)	1.262 (1.130–1.394)	0.097 (0.057–0.157)	0.128 (0.062–0.219)	0.883 (0.753–1.013)	2.578 (1.491–4.171)	3.420 (1.663–5.913)	0.935 (0.798–1.072)
South Asia	0.282 (0.222–0.390)	0.573 (0.344–0.722)	2.278 (2.158–2.398)	0.245 (0.191–0.342)	0.395 (0.241–0.497)	1.500 (1.318–1.682)	5.938 (4.665–8.213)	9.362 (5.642–11.938)	1.396 (1.202–1.591)
Southeast Asia	0.247 (0.162–0.303)	0.490 (0.260–0.614)	2.445 (2.309–2.581)	0.194 (0.126–0.238)	0.291 (0.154–0.362)	1.496 (1.323–1.669)	4.944 (3.158–6.095)	7.470 (3.893–9.387)	1.505 (1.341–1.670)
Southern Latin America	0.567 (0.469–0.683)	0.712 (0.587–0.839)	0.546 (0.173–0.920)	0.383 (0.318–0.465)	0.340 (0.284–0.396)	−0.583 (−0.953 to −0.212)	9.433 (7.851–11.259)	8.208 (6.850–9.478)	−0.693 (−1.061 to −0.323)
Southern sub‐Saharan Africa	0.564 (0.423–0.768)	0.936 (0.659–1.199)	1.570 (1.193–1.949)	0.443 (0.332–0.610)	0.633 (0.448–0.795)	1.076 (0.617–1.538)	11.433 (8.612–15.288)	15.823 (11.211–20.466)	0.920 (0.444–1.399)
Tropical Latin America	0.279 (0.256–0.299)	0.695 (0.638–0.754)	3.058 (2.635–3.481)	0.202 (0.186–0.218)	0.369 (0.338–0.396)	2.038 (1.621–2.457)	5.335 (4.941–5.729)	9.880 (9.093–10.579)	2.014 (1.595–2.435)
Western Europe	0.475 (0.442–0.507)	0.880 (0.775–0.976)	1.815 (1.260–2.374)	0.225 (0.210–0.238)	0.245 (0.219–0.269)	−0.124 (−0.656–0.410)	5.343 (4.994–5.689)	5.960 (5.329–6.606)	−0.034 (−0.580–0.515)
Western sub‐Saharan Africa	0.488 (0.283–0.740)	0.496 (0.317–0.874)	−0.141 (−0.243 to −0.038)	0.434 (0.253–0.652)	0.390 (0.249–0.677)	−0.544 (−0.635 to −0.453)	10.474 (6.110–16.084)	9.092 (5.942–16.465)	−0.689 (−0.802 to −0.576)

During the previous three decades, the global number of MBC‐associated deaths has risen by 1.710 times (4899.024; 95% UI, 4142.232–5895.072) in 1990 vs. 13274.114 (95% UI, 9074.300–16240.064) in 2021 (Table 1). Similarly, the MBC‐associated ASDR increased from 0.282 (95% UI, 0.241–0.338) in 1990 to 0.335 (95% UI, 0.232–0.409) in 2021; the EAPC was 0.664 (95% CI, 0.562–0.767; Table 2).

Additionally, the global number of MBC‐associated DALYs increased by 1.628 times (144956.307; 95% UI, 121255.738–172477.024) in 1990 vs. 380916.567 (95% UI, 252900.074–476416.749) in 2021 (Table 1). Consistently, the ASDALYR increased from 7.386 (95% UI, 6.230–8.827) in 1990 to 9.157 (95% UI, 6.116–11.423) in 2021; the EAPC was 0.853 (95% CI, 0.750–0.956; Table 2). Over the past three decades, the overall MBC burden has been on the rise.

### 3.2 Geographic Regional Burden

**FIGURE 1:**
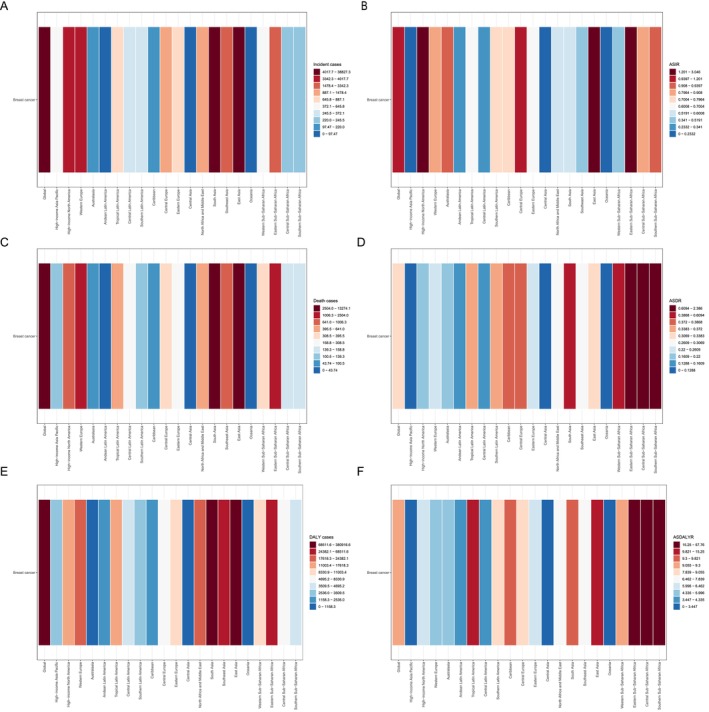
Cases and age‐standardized rates of male breast cancer incidence, death, and DALY in global and 21 geographic regions. (A) Incident cases: Regional variations in MBC incidence, with East Asia reporting the highest cases and Oceania the lowest. (B) AISR: Differences in AISRs across regions, with the highest in Eastern sub‐Saharan Africa and the lowest in Oceania. (C) Death cases: East Asia recorded the highest death cases, whereas Oceania reported the lowest. (D) ASDR: Regional disparities in ASDRs, with the highest in Eastern sub‐Saharan Africa and the lowest in high‐income Asia Pacific. (E) DALY cases: East Asia showed the highest DALY burden, whereas Oceania had the lowest. (F) ASDALYR: The highest burden rate was observed in Eastern sub‐Saharan Africa, whereas high income Asia Pacific had the lowest.

Among 21 geographic regions, East Asia had the most incident cases of MBC in 2021 (17122.003; 95% UI, 6888.342–24713.833), whereas Oceania had the fewest (7.387; 95% UI, 3.549–12.522; Figure 1A and Table 1). Eastern sub‐Saharan Africa experienced the highest ASIR of MBC (3.046; 95% UI, 2.009–5.461), whereas Oceania had the lowest (0.180; 95% UI, 0.087–0.305) in 2021. From 1990 to 2021, the ASIR of MBC exhibited the largest increase in East Asia (EAPC, 6.227; 95% CI, 5.457–7.003) and the largest decrease in Eastern Europe (EAPC, −1.253; 95% CI, −1.551 to −0.954; Figure 1B and Table 2).

In addition, East Asia experienced the most MBC death cases in 2021 (3439.080; 95% UI, 1368.006–4948.593), whereas Oceania had the fewest (4.859; 95% UI, 2.352–8.454; Figure 1C and Table 1). The ASDR of MBC was highest in Eastern Sub–Saharan Africa (2.386; 95% UI, 1.601–4.286). In contrast, the ASDR of MBC was lowest in the High‐income Asia Pacific (0.063; 95% UI, 0.058–0.069) in 2021. From 1990 to 2021, the largest ASDR increase of MBC was in Central Asia (EAPC, 3.792; 95% CI, 2.929–4.662), whereas the largest decrease was in Eastern Europe (EAPC, −2.460; 95% CI, −2.806 to −2.112; Figure 1D and Table 2).

In addition, East Asia had the most DALY cases of MBC in 2021 (110295.773; 95% UI, 42489.925–159208.325), whereas Oceania had the fewest (154.077; 95% UI, 73.038–266.404; Figure 1E and Table 1). Eastern sub‐Saharan Africa had the highest ASDALYR of MBC (57.763; 95% UI, 37.899–105.577), whereas high‐income Asia Pacific had the lowest (1.493; 95% UI, 1.339–1.666). From 1990 to 2021, Central Asia had the largest increase in the ASDALYR of MBC (EAPC, 3.489; 95% CI, 2.675–4.310), whereas Eastern Europe had the largest decrease (EAPC, −2.384; 95% CI, −2.753 to −2.013; Figure 1F and Table 2).

### 3.3 Different Countries and Territories Burden

**FIGURE 2:**
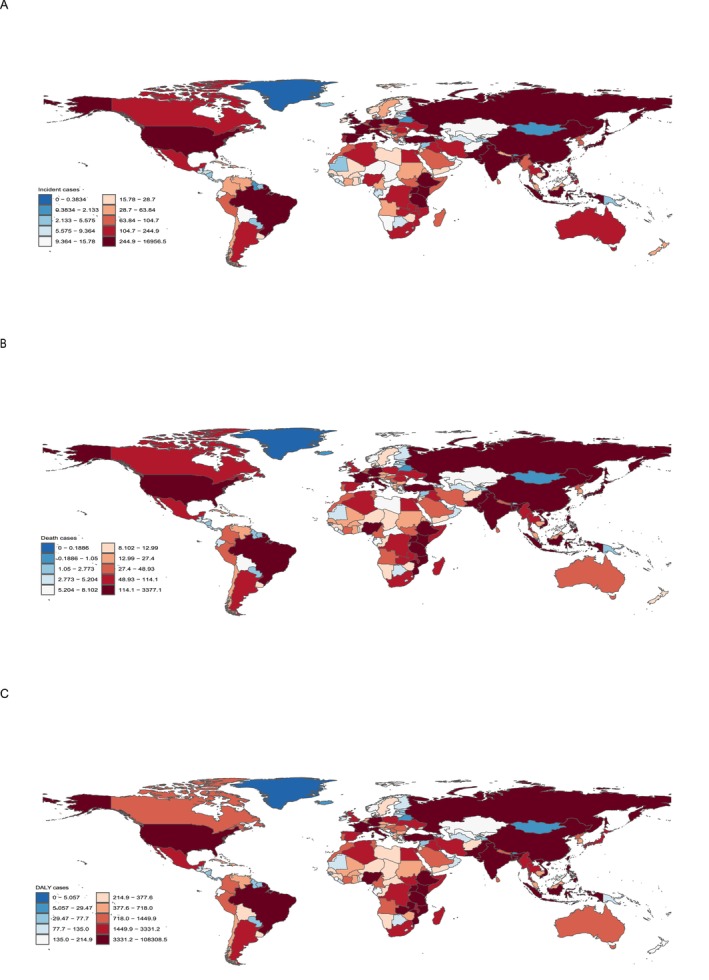
Male breast cancer incident, death, and DALY cases in 204 countries and territories. (A) Incident cases: China reported the highest number of cases (16,956; 95% UI: 6780–24,541), whereas Tokelau reported the lowest (0.002; 95% UI: 0.001–0.003); (B) Death cases: The highest number of deaths occurred in China (3377; 95% UI: 1326–4894), and the lowest in Tokelau (0.001; 95% UI: 0.001–0.002); (C) DALY cases: China also had the highest DALY burden (108,308; 95% UI: 41,297–157,050), whereas Tokelau had the lowest (0.035; 95% UI: 0.019–0.055).

**FIGURE 3:**
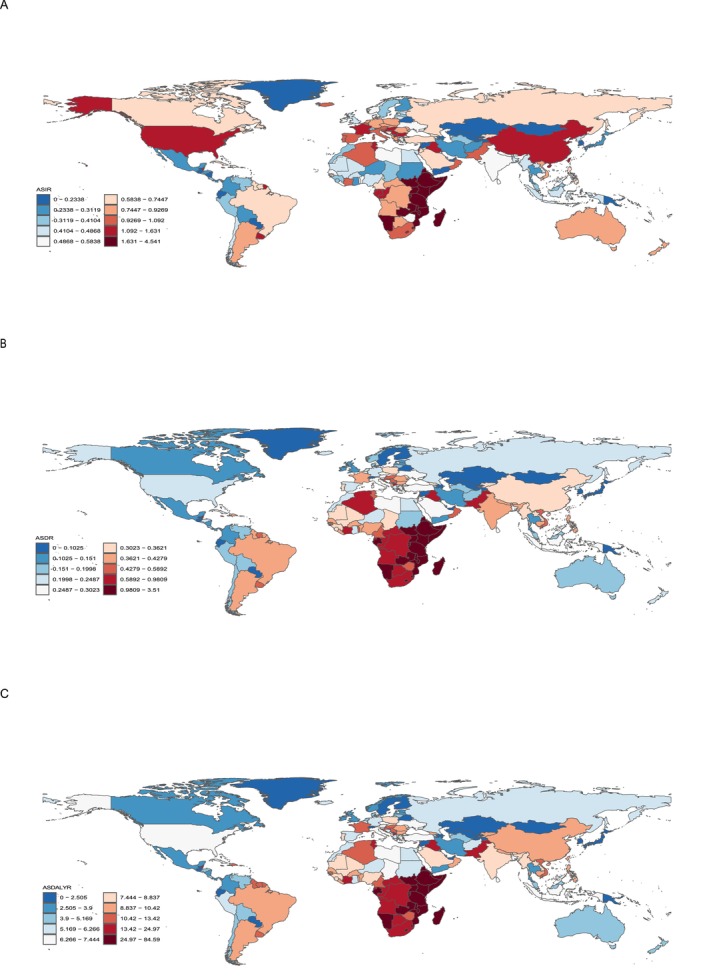
Age‐standardized rates of male breast cancer incidence, death, and DALY per 100,000 population in 204 countries and territories. (A) AISR: Uganda had the highest ASIR (4.541; 95% UI: 3.028–6.808), whereas Belarus had the lowest (0.022; 95% UI: 0.016–0.029); (B) ASDR: Uganda reported the highest ASDR (3.510; 95% UI: 2.301–5.195), and Belarus the lowest (0.007; 95% UI: 0.005–0.008); (C) ASDALYR: Uganda had the highest ASDALYR (84.592; 95% UI: 55.883–129.235), whereas Belarus had the lowest (0.205; 95% UI: 0.158–0.260).

In 2021, among 204 countries and territories, China had the most incident (16956.479; 95% UI, 6779.890–24541.319), death (3377.114; 95% UI, 1326.020–4893.848), and DALY cases (108308.473; 95% UI, 41296.692–157049.583). Meanwhile, Tokelau had the fewest incident (0.002; 95% UI, 0.001–0.003), death (0.001; 95% UI, 0.001–0.002), and DALY cases (0.035; 95% UI, 0.019–0.055; Figure 2 and Table S1). Moreover, Uganda had the highest ASIR (4.541; 95% UI, 3.028–6.808), ASDR (3.510; 95% UI, 2.301–5.195), and ASDALYR (84.592; 95% UI, 55.883–129.235). Nevertheless, Belarus had the fewest ASIR (0.022; 95% UI, 0.016–0.029), ASDR (0.007; 95% UI, 0.005–0.008), and ASDALYR (0.205; 95% UI, 0.158–0.260). From 1990 to 2021, Georgia had the largest increase in ASIR (EAPC, 35.196; 95% CI, 28.924–41.772), ASDR (EAPC, 35.120; 95% CI, 28.794–41.757), and ASDALYR (EAPC, 29.285; 95% CI, 24.397–34.364). However, Belarus had the largest decrease of ASIR (EAPC, −8.645; 95% CI, −12.308 to −4.828), ASDR (EAPC, −10.099; 95% CI, −13.811 to −6.226), and ASDALYR (EAPC, −9.593; 95% CI, −13.212 to −5.824; Figure 3 and Table S2).

### 3.4 Age Burden and Trend Changes

**FIGURE 4:**
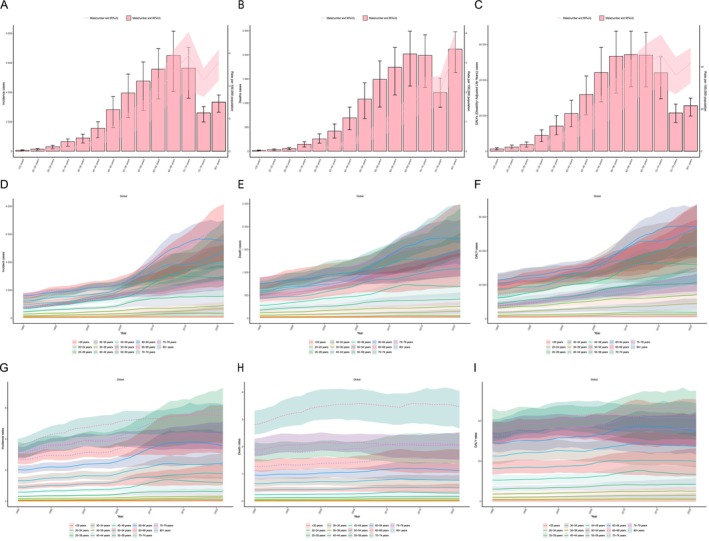
Age‐structured analysis of male breast cancer burden in 2021 and global temporal trends, 1990–2021. Age‐structured analysis of (A) incident, (B) death, and (C) DALY cases and rates. Global temporal trends of (D) incident, (E) death, and (F) DALY cases. Global temporal trends of (G) AISR, (H) ASDR, and (I) ASDALYR.

In 2021, the incident, death, and DALY cases all increased with age, with the highest incident case number being observed in the 65–69 age group (6491.678; 95% UI, 4059.763–8144.690). The 70–74 age group had the most significant incidence rate (5.856; 95% UI, 3.736–7.304; Figure 4A). The over 80 age group experienced the highest death case number (2117.733; 95% UI, 1631.968–2477.136) and the highest death rate (3.458; 95% UI, 2.665–4.045; Figure 4B). The 60–64 age group had the highest DALY case number (54363.372; 95% UI, 36272.640–67457.737), whereas the 70–74 age group had the highest DALY rate (45.792; 95% UI, 30.613–55.092; Figure 4C).

From 1990 to 2021, the incident case number has steadily increased, with the most significant rise observed in the 70–74 and 80+ age groups. Incidence rates are highest among older adults, particularly those aged 60 years and above, indicating a significant age‐related increase in disease burden (Figure 4D,G). Similarly, death cases have risen over time, especially among those aged 60 years and above. The 80+ age group had the highest death rates, underscoring a significant age‐related increase in mortality (Figure 4E,H). Additionally, there has been a significant increase in DALY cases over this period, particularly in the 60–64 and 65–69 age groups. The DALY rates peak in the 70–74 age group, highlighting a significant age‐related increase in disability and life years lost (Figure 4F,I).

### 3.5 RFs for MBC

**FIGURE 5:**
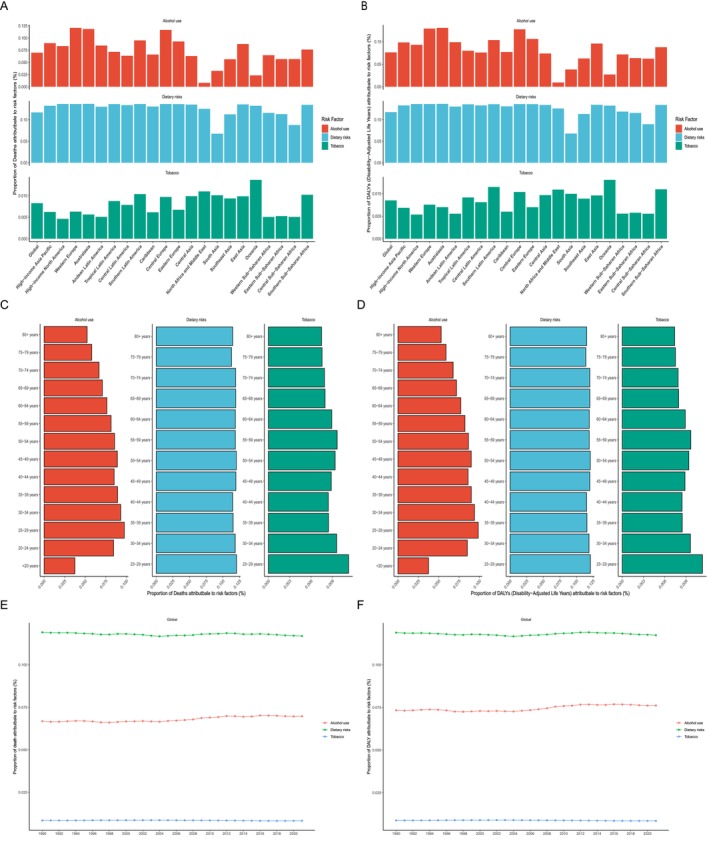
Risk factors and temporal trends of male breast cancer by age and 21 geographic regions. The proportion of (A), (C), and (E) deaths, as well as (B), (D), and (F) DALYs attributable to risk factors in 21 geographic regions, different age groups, and global temporal trends, respectively.

The GBD database identifies three key RFs for MBC: alcohol use, dietary risks, and tobacco. On a global scale, dietary risk was the most important attributable RF for MBC deaths, with a death percentage of 11.690% (95% UI, −0.003%–24.838%). Among the 21 geographical regions, the highest proportion was in Australasia at 13.674% (95% UI, −0.006%–28.990%), whereas the lowest was in South Asia at 6.743% (95% UI, −0.001%–14.773%; Figure 5A). Consistently, dietary risks were responsible for 11.739% of MBC‐associated DALYs globally (95% UI, −0.003%–24.938%), with the highest proportion also in Australasia at 13.667% (95% UI, −0.007%–28.953%) and the lowest in South Asia at 6.780% (95% UI, −0.001%–14.965%; Figure 5B). The proportion of deaths and DALYs attributable to dietary risks remains relatively stable across all age groups, indicating minimal variation in the impact of dietary risks on health outcomes across different ages (Figure 5C,D). From 1990 to 2021, dietary risks have always been attributed to the highest proportion of deaths or DALYs, followed by tobacco and alcohol use, with relatively stable trends over the period (Figure 5E,F).

## 4 Discussion

In recent decades, MBC incidence has shown a notable increase worldwide. The increasing incidence is coupled with a higher death rate in males compared to females, primarily due to later‐stage diagnoses. This trend, along with the associated medical and social costs, underscores the urgency of addressing MBC as a significant public health issue. Here, we investigated MBC‐associated incidence, death, and DALY cases with corresponding ASRs in all GBD regions and countries throughput 1990–2021. Our findings provide insights regarding the MBC burden over the past three decades in different regions and countries. Our results reinforce findings from previous studies, which indicated that the burden of MBC is increasing in some regions and countries worldwide [15, 16, 17]. A global assessment of the epidemiologic patterns of MBC may help policymakers and clinicians to develop appropriate prevention and management strategies.

In 2021, the global MBC incident cases were 38,827.302 (95% UI, 24,650.482–47,845.887), with an ASIR of 0.941 (95% UI, 0.605–1.155), and the corresponding death cases reached 13,274.114 (95% UI, 9074.300–16,240.064), with an ASDR of 0.282 (95% UI, 0.241–0.338). Additionally, the global DALY cases were 380,916.567 (95% UI, 252,900.074–476,416.749), with an ASDALYR of 9.157 (95% UI, 6.116–11.423; Tables 1 and 2). Nevertheless, it is important to interpret these data with caution because of the broad UI, indicating significant uncertainty in these estimations. The observed rise in data could perhaps be attributed to population expansion or improvements in diagnostic capabilities [18, 19]. However, additional research is needed to fully comprehend the influence of these factors on MBC epidemiology. From 1990 to 2021, the ASIR, ASDR, and ASDALYR of MBC indicate an increasing trend globally. This suggests that the burden of MBC is not only increasing in terms of new cases but also in mortality and years of life lost due to disability, reflecting a more comprehensive global health impact. This trend aligns with findings from other studies indicating a broad increase in MBC incidence, partly driven by enhanced screening programs and heightened awareness [2, 15, 17]. However, the simultaneous rise in ASDR and ASDALYR suggests that improvements in early detection alone may not be sufficient to mitigate the overall burden. Factors such as population aging, lifestyle changes, and environmental exposures may have contributed to the rising mortality and disability associated with MBC [20, 21]. These observations highlight the importance of ongoing assessment and enhancement of MBC preventive and control methods to ensure they are in line with current population dynamics and healthcare practices.

Although there have been worldwide endeavors in healthcare and cancer prevention, we have observed a significant disparity in MBC burden trends regionally and nationally. Out of the 204 countries and territories, some recognized ones are Uganda, Mozambique, Zambia, Malawi, and South Sudan. The majority of these nations are economically underdeveloped, resulting in a significant disease burden measured in terms of incidence, mortality, and DALYs in 2021, because of the lack of adequate social support and effective environmental governance. Geographical disparities in MBC burden can also be attributed to factors such as limited access to healthcare facilities and early diagnostic services, low public awareness about MBC, and socioeconomic challenges that delay medical consultation and treatment [22]. Additionally, environmental factors and a higher prevalence of RFs, such as HIV infection and exposure to carcinogens, may contribute to the increased rates observed in these areas [23]. Regarding nations witnessing a decline in ASRs of incidence, mortality, or DALYs from 1990 to 2021, most of these countries are classified as high‐income economies. These countries include Republic of Korea, Finland, Switzerland, Singapore, and United Kingdom. The reduction in ASRs in these regions is largely attributable to the implementation of robust, resilient, and easily accessible healthcare systems, such as Europe's Beating Cancer Plan [24]. This plan is a fundamental part of the new initiative and aims to support member states in their endeavors to prevent cancer and improve the well‐being of cancer patients, survivors, and their families and caregivers [25, 26]. These disparities highlight the need for region‐specific strategies that address the unique challenges faced by different countries in MBC management and prevention. Notably, considering the multifaceted nature of healthcare systems and the intricate interaction of socioeconomic, environmental, and public health factors are essential when interpreting changes in disease incidence rates [27, 28].

This study also examined the age disparities in the disease burden of MBC. Research indicates that the prevalence of MBC increases with advancing age, with males aged 60 years or older accounting for approximately 75% of all cases [2]. Our findings show that from 1990 to 2021, there has been a significant age‐related increase in the burden of MBC, with notably higher incidence and mortality rates observed among those aged 60 years and above. The substantial rise in DALY cases, particularly among men aged 60–74, underscores the growing disability and life years lost due to this disease. Older men often face significant challenges because of higher rates of comorbidities, such as cardiovascular diseases and diabetes, which complicate treatment and increase DALY rates [29]. Additionally, disparities in healthcare access, including mobility limitations and financial constraints, further delay diagnosis and treatment [30]. These findings underscore the urgent need for targeted screening, early detection, and age‐specific treatment strategies to improve outcomes and reduce the impact of MBC in older populations.

Herein, tobacco, alcohol use, and dietary hazards were identified as significant RFs for MBC. These factors contribute to a proportion of the MBC burden, which aligns with findings from prior studies [31, 32, 33]. Moreover, MBC development has been associated with exogenous estrogen, obesity, alcohol usage, liver disease, and Klinefelter syndrome [2, 34, 35, 36]. The interaction between these RFs can compound the overall risk of MBC. For instance, alcohol consumption and tobacco use have been shown to have a synergistic effect on increasing cancer risk due to their combined impact on estrogen levels and the immune system. Alcohol can increase estrogen levels in the body, whereas tobacco contains carcinogens that may damage breast tissue [37, 38]. When combined with poor dietary habits, such as high‐fat intake or low consumption of fruits and vegetables, these factors can further exacerbate inflammation and oxidative stress, creating an environment conducive to cancer development [39]. This interaction suggests that individuals exposed to multiple RFs may have a significantly higher risk of developing MBC compared to those with a single risk factor. Obesity raises MBC risk due to its correlation with elevated estrogen levels, decreased testosterone levels, and reduced levels of sex hormone‐binding globulin. These factors are involved in increased estrogen bioavailability, which further enhances MBC risk. This may be attributed to elevated estrogen levels and reduced testosterone levels in obese males [40]. Our analysis reveals that dietary risks have consistently been the leading cause of mortalities or DALYs from 1990 to 2021. Tobacco and alcohol consumption have followed closely behind, with very stable patterns observed over the whole time. Moreover, cancer incidence, mortality, and prevalence disparities arise from the intricate interplay of genetic factors, aging, modifiable RFs, and socioeconomic determinants [41]. Consequently, it is imperative to implement more logical and well‐reasoned public policies to regulate these RFs.

To mitigate the growing burden of MBC, valuable lessons can be drawn from successful public health policies that have effectively reduced cancer rates and mortality. For instance, Europe's Beating Cancer Plan has decreased cancer incidence across EU member states through early detection initiatives, public awareness campaigns, and promoting healthier lifestyles to reduce RFs such as tobacco and alcohol use [42]. Similarly, the World Health Organization's Framework Convention on Tobacco Control (FCTC) has significantly reduced tobacco‐related cancers globally through taxation, smoking bans, and anti‐tobacco advertising [43]. National cancer control programs in countries like the United Kingdom and Australia have also demonstrated that early detection and robust healthcare systems can markedly reduce cancer mortality [43, 44]. To reduce the burden of MBC, we recommend targeted screening for older men, especially those at higher risk due to family history or genetic mutations. This should include regular mammograms and clinical exams, along with awareness campaigns to promote early detection. Additionally, addressing cultural barriers and stigma is key. Public health campaigns should highlight that BC affects both men and women, helping to dispel misconceptions and encourage timely medical attention. These initiatives could significantly reduce MBC's impact, particularly in older populations.

There were certain constraints in this study. The accuracy of our results is contingent upon GBD data quality and quantity. The GBD research is unable to comprehensively encompass all regions of the world in terms of quantity. Regarding quality, it is possible that less developed countries may have lacking information. Furthermore, the GBD database, which we used for analysis, does not include detailed information on genetic RFs, preventing us from fully exploring the genetic components of MBC. For instance, known genetic mutations such as BRCA1 and BRCA2, which significantly increase BC risk [45], could not be analyzed in this context. Additionally, genetic conditions like Klinefelter syndrome, which are associated with an increased risk of MBC due to altered hormone levels, could not be thoroughly investigated [46]. Our investigation into the role of genetic factors on MBC was limited by a lack of information [34], preventing us from delving further into its history. Furthermore, various RFs can have distinct impacts on distinct histological subtypes of MBC [47]. However, at present, we lack the ability to accurately differentiate between these diverse histological subtypes. This limits our understanding of the role genetic predispositions play in MBC development. Future research should focus on identifying novel genetic mutations or polymorphisms and studying gene–environment interactions to provide deeper insights into MBC etiology. Expanding genomic studies in diverse populations and examining severity grading and subtypes will be crucial for developing more effective preventive interventions and targeted treatments [46].

## 5 Conclusion

Collectively, GBD 2021 offers more precise and reliable data on MBC incidence, death, and DALYs. Analyzing these data in our study demonstrates that the worldwide MBC burden continues to be significant, particularly in less developed nations and among older populations. The results indicate a substantial correlation between MBC and men's lifestyles, emphasizing the need to modify both food habits and overall lifestyle for both individuals already affected by the disease and those seeking to prevent it. Furthermore, our findings highlight the urgent need for region‐specific interventions, taking into account the unique socioeconomic and healthcare challenges faced by different regions. Accordingly, modifiable RFs, such as tobacco and alcohol use, as well as dietary habits, should be targeted through public health campaigns to reduce MBC incidence and related morbidity.

## Author Contributions

**Long Wang:** conceptualization (lead), resources (lead), writing – original draft (lead). **Ping Wen:** data curation (lead), formal analysis (lead). **Qing Shao:** funding acquisition (lead), software (lead). **Dongping Jiang:** formal analysis (lead), software (lead). **Yulan Zhao:** investigation (lead), project administration (lead), visualization (lead). **Xiaohua Zeng:** funding acquisition (lead), supervision (lead), writing – review and editing (lead).

## Ethics Statement

Obtaining and using anonymized, publically available epidemiologic data from the database does not necessitate ethical approval or patient‐informed consent.

## Conflicts of Interest

The authors declare no conflicts of interest.

## Supporting information


Table S1.

Table S2.

## Data Availability

The study data have open access through the GBD 2021 online database, as the Methods section outlined.
